# High dielectric ternary oxides from crystal structure prediction and high-throughput screening

**DOI:** 10.1038/s41597-020-0418-6

**Published:** 2020-03-06

**Authors:** Jingyu Qu, David Zagaceta, Weiwei Zhang, Qiang Zhu

**Affiliations:** 1 College of Science, China Agricultural University, Beijing, 100083 China; 2 Department of Physics and Astronomy, University of Nevada, Las Vegas, NV 89154 USA

**Keywords:** Density functional theory, Materials for devices, Electrical and electronic engineering

## Abstract

The development of new high dielectric materials is essential for advancement in modern electronics. Oxides are generally regarded as the most promising class of high dielectric materials for industrial applications as they possess both high dielectric constants and large band gaps. Most previous researches on high dielectrics were limited to already known materials. In this study, we conducted an extensive search for high dielectrics over a set of ternary oxides by combining crystal structure prediction and density functional perturbation theory calculations. From this search, we adopted multiple stage screening to identify 441 new low-energy high dielectric materials. Among these materials, 33 were identified as potential high dielectrics favorable for modern device applications. Our research has opened an avenue to explore novel high dielectric materials by combining crystal structure prediction and high throughput screening.

## Abstract (summary)

**Table:** 

Measurement(s)	high dielectric ternary oxides
Technology Type(s)	computational modeling technique • high throughput sample analysis

Machine-accessible metadata file describing the reported data: 10.6084/m9.figshare.11698986

## Background & Summary

A dielectric is an insulator that becomes polarized under the influence of an applied electric field. Materials exhibiting a high dielectric constant ($\epsilon$) are promising for energy storage applications. As an example, a parallel-plate capacitor’s energy storage capability is approximately expressed as $U=\frac{1}{2}\epsilon {\epsilon }_{0}{E}^{2}$, where $\epsilon$_0_ and $\epsilon$ are the permittivity of vacuum and the material dielectric constant respectively, and *E* is the applied electric field. Materials with high dielectric constants, compared to materials with lower dielectrics, have the potential to store more charge per unit volume, which is critical to high-performance device fabrication as well as miniaturization. Many high dielectric materials, such as ZrO_2_^1^, HfO_2_^2^, Al_2_O_3_^3,4^, Y_2_O_3_^5^, SrTiO_3_^6^ and BaTiO_3_^7^, have been extensively applied in microelectronic technologies. However, currently used dielectrics are inhibiting the development of cheaper and more efficient devices. For example, BaTiO_3_ applied to multi-layer ceramic capacitors (MLCC) encounters certain limitations concerning the continuing miniaturization of circuit components, which require thinner dielectric layers while retaining the reliability of current advanced capacitors. Decreasing the particle size of BaTiO_3_ introduces a so-called “size effect” wherein the ferroelectricity reduces with decreasing particle size and then vanishes below a specific critical size^8^. There also exists a technical challenge when implementing SiO_2_ in complementary metal–oxide–semiconductor (CMOS) and dynamic random-access memory (DRAM) devices. When the thickness of SiO_2_ is reduced to a few nanometers, leakage current increases greatly because of the quantum tunneling^9^. The bandgap (*E*_*g*_) is also a key property affecting device performance. In flash memory, a large band gap is necessary to satisfy the stringent leakage current specification (0~10^−9^ A · cm^−1^)^10^. However, there exists a general inverse correlation between $\epsilon$ and *E*_*g*_. Thus, the exploration of new high dielectrics requires a careful balance between the bandgap and dielectric constant.

To date, there are only a few hundred known materials with measured dielectric constants; this includes both organic and inorganic materials. The dielectric constants of the vast majority known inorganic compounds (~30,000) are currently unknown. Modern quantum mechanical calculations provide complementary approaches to experiments with far lower costs. In exploring new materials with high dielectric constants, high-throughput screenings of candidate materials based on density functional theory (DFT) calculations have become popular recently. Utilizing a high-throughput setting, Yim *et al*.^10^ calculated properties for more than 1800 structures of binary and ternary oxides from the Inorganic Crystal Structure Database (ICSD) and generated a total property map of band gap versus dielectric constant. Petousis *et al*.^11,12^ developed a computational infrastructure to perform high-throughput screening of dielectric materials based on Density Functional Perturbation Theory (DFPT) and constructed a database of dielectric tensors consisting of 1,056 inorganic ordered compounds. However, these studies focused on only already known materials. Recent successes in crystal structure prediction (CSP) has shown that it is possible to predict new materials prior to synthesis^13^. Sharma *et al*.^14^ designed organic polymer dielectrics using a strategy of hierarchical modeling, and their efforts led to the successful synthesis of several new high-$\epsilon$ polymers. Zeng *et al*.^15,16^ applied CSP methodology to explore high dielectrics in particular systems: hafnia-based oxides and Zr_*x*_Si_1−*x*_O. These results inspired us to explore potential high dielectrics in an expanded chemical space.

In this study, we chose to focus on finding new ternary oxides possessing both high $\epsilon$ and *E*_*g*_, given that binary oxides have already been well studied^17,18^. In this work, we combined high throughput calculations with crystal structure prediction methods to screen for target materials in a broad chemical space. Specifically, we chose chemical systems based on the combination of two types of metal oxides between group II*A*/III*A*/IV*A* and group IV*B*, namely Ca(Be, Mg, Sr, Ba)O-Ti(Hf, Zr)O_2_, Al(Ga, In)_2_O_3_-Ti(Hf, Zr)O_2_, and Si(Ge)O_2_-Ti(Hf, Zr)O_2_. For each system of *A*_*m*_O_*n*_ − *B*O_2_ (*A*: II*A*/III*A*/IV*A*; *B*: IV*B*), we performed variable composition CSP calculations to search for low energy structures which are likely to be (meta)stable if they can be synthesized. The low energy structures were then extracted and fed to our newly developed computational pipeline to screen their dielectric and band gap properties. As a result, we have provided a list of hypothetical materials which are favorable for high dielectric applications.

## Methods

### Theory and definitions

There are two mechanisms that contribute to a materials dielectric tensor: the ionic contribution which is a consequence of atomic displacement, and the electronic contribution which is a consequence of electron cloud distortion. As a result, the dielectric tensor can be represented as a sum of these two mechanisms ${\epsilon }_{\alpha \beta }={\epsilon }_{\alpha \beta }^{0}+{\epsilon }_{\alpha \beta }^{{\rm{\infty }}}$, where *α* and *β* denote the directions of the applied electric field and the resulting polarization in the Cartesian coordinate system. In most cases, the electronic contribution ${\epsilon }_{\alpha \beta }^{{\rm{\infty }}}$ is much smaller than the ionic part and can be somewhat disregarded. The ionic dielectric tensor component ${\epsilon }_{\alpha \beta }^{0}$, due to the atomic displacements in the crystalline unit cell, is much more pronounced. Following ref. ^19^, we can obtain the ${\epsilon }_{\alpha \beta }^{0}$ value by summing the contribution from each vibrational phonon mode,

1 $${\epsilon }_{\alpha \beta }^{0}=\frac{4\pi }{{\Omega }_{0}}\sum _{m}\frac{{S}_{m,\alpha \beta }}{{\omega }_{m}^{2}},$$

where ${\Omega }_{0}$ is the volume of the primitive cell, ${\omega }_{m}$ denotes the frequency of each vibration mode. ${S}_{m,\alpha \beta }$ is the mode-oscillator strength tensor and can be obtained by

2 $${S}_{m,\alpha \beta }=\left(\sum _{\kappa \alpha {\prime} }\,{Z}_{\kappa ,\alpha \alpha {\prime} }^{\ast }{U}_{m}^{\ast }\left(\kappa \alpha {\prime} \right)\right)\left(\sum _{\kappa {\prime} \beta {\prime} }\,{Z}_{\kappa {\prime} ,\beta \beta {\prime} }^{\ast }{U}_{m}\left(\kappa {\prime} \beta {\prime} \right)\right),$$

where ${Z}_{\kappa ,\alpha \beta }^{\ast }$ is the Born effective charge and ${U}_{m}(\kappa \beta )$ are eigen-displacements. The eigen-displacements form an orthonormal basis as shown in the following equation.

3 $$\sum _{\kappa \beta }\,{M}_{\kappa }{\left[{U}_{m}(\kappa \beta )\right]}^{\ast }{U}_{n}(\kappa \beta )={\delta }_{mn},$$

where ${M}_{\kappa }$ is the mass of the ion $\kappa$. It is obvious $\epsilon$ is dependent on various fundamental quantities which include: Born effective charge, ionic mass, phonon frequencies, and cell volume. A detailed analysis of the influence of these quantities on both ${E}_{g}$ and $\epsilon$ is provided in Data records section.

For the predicted stable and meta-stable structures, when they are applied to electronic devices, both dielectric constant and bandgap need to be considered. To evaluate the performance of materials, we used a fitness model proposed in a recent work^15^:

4 $$max{F}_{ED}=8.1882\,J\,{{\rm{c}}{\rm{m}}}^{-3}\times \epsilon {({E}_{g}/{E}_{gc})}^{2\alpha }$$

where *α* is 1 for an insulator and 3 for semiconductors, *E*_*g*_ is the bandgap and *E*_*gc*_ is 4 eV, which represents the critical value to distinguish semiconductors and insulators. Using this descriptor, one can estimate the level of energy storage for any given material.

### Workflow

**Fig. 1:**
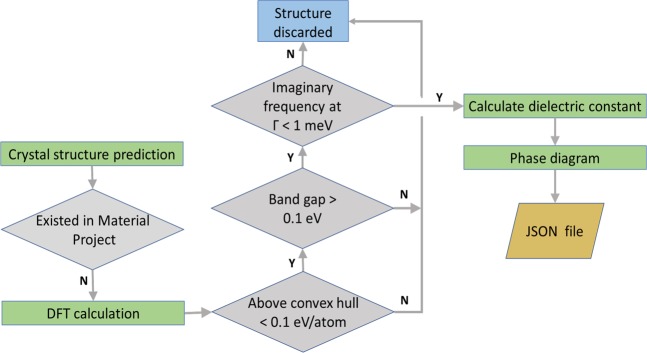
Flowchart summarizing the calculation method and process.

We performed the screening work by following the flowchart as summarized in Fig. 1. The first step is to generate new structures of *A*_*x*_O_1−*x*_-*B*O_2_ from structure prediction. The obtained structures were then compared with the Materials Project database^20^. Since the dielectric properties of structures available in the Materials Project database have been screened thoroughly, we focused on the new structures generated from CSP (based on energy optimization). The formation energy, band gap, and dielectric constants of the new structures were further calculated by DFT with higher accuracy. To avoid massive calculations, we considered only the structures satisfying the following conditions: (1) within 0.1 eV/atom from the convex hull, (2) bandgap > 0.1 eV. We then calculated the phonon spectrum based on DFPT. Those materials with imaginary frequency at Γ point (greater than 1 meV) were also discarded. Only the structures satisfying all criteria were stored for further dielectric calculation. Last, we used the fitness model mentioned in Eq. 4 to evaluate the level of energy storage of these materials.

#### Crystal structure prediction

We utilized the USPEX code^21^ to search for new materials within the chemical space described above. For each system (e.g., CaO-TiO_2_), we performed a CSP calculation with 50 individuals in each generation for 18 generations in total. We initiate the first generation of structures with structures of random composition and space group. The structures in the subsequent generations were generated according to the following variation operators heredity (30%), random (30%), mutation (20%) and permutation (20%), respectively. The maximum number of atoms in the unit cell is constrained to 24.

#### Structure optimization in DFT

For each structure generated from CSP, DFT calculation was performed with the Vienna ab intio software package (VASP)^22^, using the all-electron projector wave (PAW) method^23,24^. The exchange-correlation energy is treated with the generalized gradient approximation (GGA) within the Perdew-Burke-Ernzerhof (PBE) framework^25^. Preparation of input parameters and data extraction were done by the Python Materials Genomics (Pymatgen) package^26^. The structures were fully relaxed until the stress tensor is less than 3 kbar. The fitness function defined in our structure prediction is the negative of the ab initio free energy of the locally optimized structure. The formation energy for each structure is determined with respect to the most stable structure of the pure elemental solids. The formation energy versus chemical composition forms convex hull. In Materials Project, a phase diagram is constructed by comparing the relative thermodynamic stability of phases belonging to the system using an appropriate free energy model (with the corrections for gases, liquids and elements^27^), the convex hull is taken to construct the phase diagram^28,29^. In our work, we combined our results and structures from Materials Project to compute the convex hull via Pymatgen, this is reasonable since we used the same functional as in Materials Project. It was found that observed metastable phases are usually not more than 0.1–0.2 eV/atom higher in energy than the ground-state structure^30^. Metastable structures may exist at ambient conditions by means of specific techniques such as doping^9^ or in nano crystalline states^31^. Herein structures with fitness values higher than 0.1 eV were discarded. All of the obtained structures were fully relaxed until the interatomic forces and the total energies (per atom) are smaller than 0.01 eV/Å and 10^−6^ eV respectively. The plane-wave basis sets have a kinetic energy cutoff of 600 eV. The grid density of k-mesh by reciprocal volume was set to be 200 Å^−3^ in Pymatgen.

#### Dielectric constant and bandgap calculation

The DFPT methodology, as implemented in the VASP code^32^ was applied to predict dielectric constants. This method has been widely used to calculate dielectric constants^11,12^ and refractive indices^33^. The dielectric tensor is composed of two parts: electronic and ionic contributions. Since the dielectric response calculated by DFPT corresponds only a monocrystalline material, for simplicity, we adopted the same approximation method proposed in Petousis’ work^11^, in which the polycrystalline dielectric constant was estimated by averaging the eigenvalues of a monocrystalline dielectric tensor. A large bandgap is another important indicator when selecting industrial high dielectrics. The reported material bandgaps were calculated through DFT using the generalized gradient approximation (GGA). It is worth mentioning that a K-point mesh of high density is required for calculation of $\epsilon$, so the K-point reciprocal density is set to be 300 Å^−3^ in Pymatgen, which is sufficient to reach the required computation accuracy. It is also worth nothing that GGA and LDA underestimate the band gap of oxides^34,35^, usually a GGA + U method is supposed to be implemented to reduce the error. The determination of U parameter is critical and usually achieved by an empirical way. In Pymatgen, U parameters are only available for a few oxides and fluorides. Here we use default setting of Pymatgen, the U parameters are not applied to our studied systems.

## Data Records

### File format

The input files containing important parameters and calculation results are stored in a JSON file, which has been uploaded to figshare^36^ and Materials Cloud^37^. For each material, one can check the properties by accessing values through keys, such as “e_poly”, “e_total” and “e_electronic”, corresponding to polycrystalline dielectric, total dielectric tensor and electronic contribution tensor, respectively (referred to Table 1). Other parameters can be found by accessing the “meta” key as listed in Table 2.

**Table 1:** Description of data keys in JSON file.

Key	Datatype	Description
e_total	array	total dielectric tensor
e_electronic	array	electronic contribution of dielectric tensor
e_poly	numeric	dielectric constant of polycrystalline
bornchrg	list	Born effective charge of each ion
mode	list	frequency, IR intensity and dielectric constant of each mode
eigenvalue	list	eigenvalues of the dynamical matrix
eigenvector	list	eigenvectors of the dynamical matrix

**Table 2:** Description of metadata keys in JSON file.

Key	Datatype	Description
space group number	numeric	space group number of structure
point group	numeric	point group of structure
bandgap	numeric	bandgap (*E*_*g*_) of material
E_formation	numeric	calculated formation energy of structure
E_above_hull	numeric	energetic distance to the convex hull
K_density	numeric	density of K points in dielectric calculation
POSCAR	string	crystal structure in VASP format
formula	string	chemical formula and multiplicative factor
POTCAR	string	pseudopotential used in VASP
INCAR	string	parameter of dielectric calculation
K_path	string	kpoint path in first brillouin zone
Kpoints	string	KPOINTS file of bandgap calculation
MP_ID	string	Materials project ID of compound

### Effect of cations on *E*_*g*_ and ${\boldsymbol{\epsilon }}$

Though there exist many factors which may affect a material’s dielectric properties, we have inferred a general trend by analyzing the data from our simulation. In finding this trend, we chose a few groups of structures belonging to the same prototype with different chemical substitutions of metal cations. In a ternary oxide *A*_*m*_O_*n*_ − *B*O_2_ (*A*: I*A*/II*A*/III*A*; *B*: IV*B*) system, we considered the roles of *A* and *B* sites separately.

**Fig. 2:**
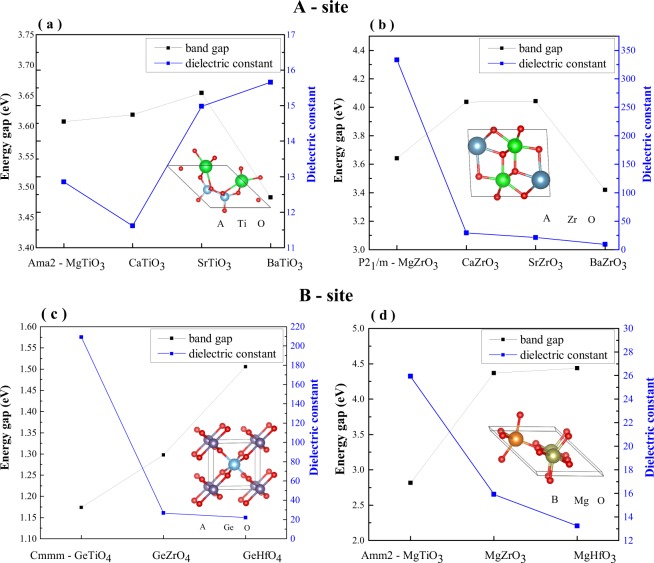
Dielectric constants and bandgap plots of different sets of compounds. (**a**) $Ama2$ II*A*-TiO_3_ (**b**) P2_1_/*m* (**c**) $Cmmm$ Ge*B*O_4_ and (**d**) $Amm2$ Mg*B*O_3_ structures (*B* = Ti, Zr, Hf). II*A*-ZrO_3_ (II*A* = Mg, Ca, Sr, Ba).

For the *A* site, we chose two groups of materials, $Ama2$ II*A*-TiO_3_ and P2_1_/*m* II*A*-ZrO_3_. As seen in Fig. 2a,b there is not any obvious correlation between the band gap and the dielectric constant for these two groups of materials. In Fig. 2a, II*A*-TiO_3_ (*A* = Mg, Ca, Sr, Ba) the reported structures are orthorhombic. The bandgap increases as the cation atomic number increases from Mg to Sr and then drops down for the Ba cation. A similar trend of bandgap can also be found in Fig. 2b. The dielectric constants in both groups follow completely different trends. This indicates that the factors affecting dielectric tensor are complicated. A more in-depth discussion of this complexity will follow.

For the *B* site, the dielectric constant and band gap of Ti/Zr/Hf-based oxides share a similar trend regardless of the difference of the cation. In Fig. 2c, three materials – GeTiO_4_, GeZrO_4_ and GeHfO_4_ share the same prototypical structure in orthorhombic $Cmmm$ symmetry. Their bandgaps increase as the atomic radius increases; however, the dielectric constants follow an inverse trend. This phenomenon was also verified by the comparison of three orthorhombic $Amm2$ Mg*B*O_3_ (*B* = Ti, Zr, Hf) structures in Fig. 2d. It is worth noting that dielectric constant of GeTiO_4_ (209.11) differ significantly from GeZrO_4_ (26.63) and GeHfO_4_ (21.80) in spite of their structural similarity, demonstrating that the composition of materials can easily influence polarization.

To better understand the atomic mechanism, we analyzed the primary contribution due to ionic vibration for all three materials in Ge*B*O_4_. From Eq. 2, we know the ionic dielectric contribution is not only related to mode frequency but also the Born effective charges (Z^*^), which are listed in Table 3 for Ge*B*O_4_ compounds. The Z^*^ values for Ge in all compounds are larger than their nominal charges (+4 a.u.), indicating the mixed covalent and ionic character of Ge atoms^38^. The decrease of Z^*^ values of Ge as the increase of the atomic number of *B* indicates the covalent character of Ge atoms is in order of GeTiO_4_ > GeZrO_4_ > GeHfO_4_. As for the *B*-site, the extraordinarily large value of Z^*^ indicates the strong covalent bond with oxygen atoms. For GeZrO_4_ and GeHfO_4_, the values of Z^*^ for Zr and Hf are very similar while Ti atoms have notably larger Z^*^ values. In essence, there is a general trend that Z^*^(Ti) > Z^*^(Zr) > Z^*^(Hf). The largest value of Z^*^ appears at Ti atom in GeTiO_4_. Xie^38^ *et al*. studied Born effective charges in perovskite Ba*M*O_3_ (*M* = Ti, Zr, Hf, Sn) and reported the same variation trend on both bandgap and Born effective charge. The variation of Born effective charge can be attributed to the dynamic charge transfer between the transition metal *d* orbitals and oxygen 2*p* orbitals by analyzing the deformation of Maximally-Localized Wannier Functions^38^.

**Table 3:** The computed Born effective charges in *Cmmm* Ge*B*O_4_.

Atom	GeTiO_4_			GeZrO_4_			GeHfO_4_		
	11	22	33	11	22	33	11	22	33
Ge	5.78	4.54	4.45	5.31	3.98	4.66	5.13	3.86	4.65
*B* (Ti, Zr, Hf)	3.85	5.85	7.77	4.58	5.70	6.21	4.54	5.55	5.91
O1	−1.35	−3.91	−2.26	−1.37	−3.53	−2.31	−1.35	−3.39	−2.29
O2	−3.46	−1.29	−3.84	−3.58	−1.32	−3.13	−3.48	−1.32	−2.99

**Fig. 3:**
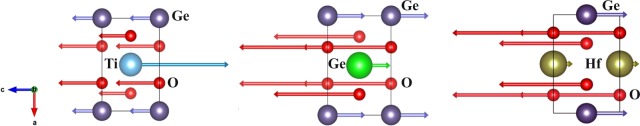
Representative vibrational modes and their contributions to the total dielectric constants in different *Cmmm*-Ge*B*O_4_ materials. (**a**) $Cmmm$-GeTiO_4_ (frequency of 55 cm^−1^), (**b**) GeZrO_4_ (frequency of 275 cm^−1^) and (**c**) GeHfO_4_ (frequency of 297 cm^−1^), respectively.

To illustrate the components of the dielectric tensor at an atomic level, we chose $Cmmm$-Ge*B*O_4_ as an example to analyze the dielectric contribution of each vibration mode in Table 4. $Cmmm$-Ge*B*O_4_ has 6 atoms in the primitive cell, which results in 18 phonon branches, of which only 8 infrared (IR) active modes have non-zero mode-oscillator strength and contribute to the ionic dielectric constant. In GeTiO_4_, the acoustic modes are dominated by the vibration of the heaviest atom, Ge with a mass (*M*) of 73. The large dielectric constant of GeTiO_4_ is mainly due to the lowest-frequency IR mode at 55 cm^−1^, which is caused by low-frequency vibration of Ti atoms along *c* axis (as seen in Fig. 3a). However, this mode is absent in GeZrO_4_ and GeHfO_4_, because the heavy Zr (*M* = 91) and Hf (*M* = 178) atoms contribute to the acoustic mode. The other modes for GeZrO_4_ and GeHfO_4_ have much higher frequencies, see Fig. 3b,c as examples, which only contribute slightly to dielectric constants. Combined with the large value of ${Z}_{33}^{* }$ of Ti, the factors including light weight, large displacement, and large Born effective charges of the Ti atom, collectively distinguish GeTiO_4_ from other materials in the prototype. Rignanese *et al*.^39^ also found that the variation in Ti oxides differs distinctively from Hf and Zr oxides and can be attributed to the difference in interatomic force constants. Similar information has been listed for all structural entries reported in this work. We believe such comprehensive analysis can yield a better understanding of the structure-dielectric property relation.

**Table 4:** The computed ionic contribution to static dielectric constants decomposed to each IR active mode (cm^−1^) in *Cmmm* Ge*B*O_4_.

GeTiO_4_				GeZrO_4_				GeHfO_4_			
Mode	${{\boldsymbol{\epsilon }}}_{{\bf{11}}}$	${{\boldsymbol{\epsilon }}}_{{\bf{22}}}$	${{\boldsymbol{\epsilon }}}_{{\bf{33}}}$	Mode	${{\boldsymbol{\epsilon }}}_{{\bf{11}}}$	${{\boldsymbol{\epsilon }}}_{{\bf{22}}}$	${{\boldsymbol{\epsilon }}}_{{\bf{33}}}$	Mode	${{\boldsymbol{\epsilon }}}_{{\bf{11}}}$	${{\boldsymbol{\epsilon }}}_{{\bf{22}}}$	${{\boldsymbol{\epsilon }}}_{{\bf{33}}}$
573	2.1	0	0	554	2.4	0	0	556	2.2	0	0
507	0	3.0	0	456	0	3.7	0	480	0	3.3	0
493	0	0	0.6	419	0	0	0.3	446	0	0	0.3
361	0	0.5	0	320	4.5	0	0	316	7.1	0	0
259	0	0	22.0	275	0	0	24.1	312	0	1.2	0
238	22.6	0	0	268	7.9	0	0	297	0	0	18.1
222	0	33.5	0	202	0	22.1	0	246	3.9	0	0
**55**	0	0	**524.8**	149	0	0	0.1	200	0	15.3	0
${\epsilon }_{0}$	24.7	37.0	547.5	${\epsilon }_{0}$	14.80	25.8	24.5	${\epsilon }_{0}$	13.2	19.8	18.4
${\epsilon }_{{\rm{\infty }}}$	5.4	5.9	7.0	${\epsilon }_{{\rm{\infty }}}$	4.7	4.8	5.3	${\epsilon }_{{\rm{\infty }}}$	4.5	4.5	5.0

### High dielectrics

**Fig. 4:**
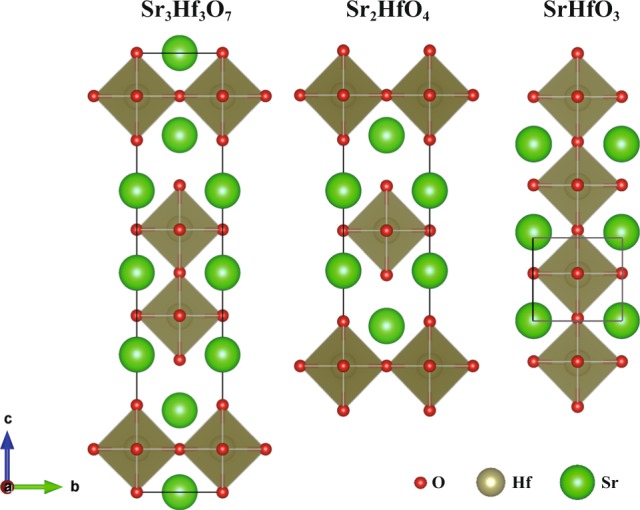
Representative high dielectric materials. Crystal structures of $I4/mmm$-Sr_3_Hf_2_O_7_ (ID = mp − 779517), $I4/mmm$-Sr_2_HfO_4_ (ID = mp − 768305) and $P4mm$-SrHfO_3_ (ID = mp − 13108), with dielectric constants of 135.00, 246.36 and 159.05, respectively, and bandgap of 3.68, 3.73 and 3.68 eV, respectively.

Our primary goal is to find a material with *E*_*g*_ and $\epsilon$ larger than BaTiO_3_, the leading material used in MLCC industry. BaTiO_3_ transforms from the high-temperature paraelectric cubic phase ($Pm$-$3m$) to the low-temperature ferroelectric tetragonal phase ($P4mm$) at 406 K^7^. The tetragonal polymorph has better ferroelectric, piezoelectric, and thermoelectric properties; hence, it is the most widely used polymorph in industry. Though the dielectric tensor is strongly dependent on temperature, we limited our study to materials at 0 K for simplicity. Most of the structures have both larger bandgaps and higher dielectric constants than tetragonal BaTiO_3_ (${E}_{g}$ = 1.72 eV, $\epsilon$ = 17.76). Among them, $I4/mmm$-Sr_3_Hf_2_O_7_ achieves the highest fitness value ($\epsilon$ = 522.12 and ${E}_{g}$ = 3.68 eV). This hypothetical structure is also available in the open Materials Project (MP) database (mp − 779517) without reported values on dielectric constants. However, after we checked the phonon, this structure is evidenced to be dynamically unstable due to the existence of imaginary phonon at X (1/2, 1/2, 0) and P (1/2, 1/2, 1/2) points. Similar conclusions were also mentioned in a recent study^40^. Further check the Sr-Hf-O system in MP database, two other structures, *P*4*mm*-SrHfO_3_ and *I*4/*mmm*-Sr_2_HfO_4_ also stand out, with the dielectric constants of 246.36 and 159.05, respectively. Their crystal structures are very similar, as shown in Fig. 4. They both crystallize into tetragonal and have Hf centered octahedra. Their bonding length (2.06 Å, 2.07 Å and 2.06 Å for Hf-O) and bandgap are almost the same. This result raises the possibility of looking for high dielectrics in these kinds of structures. In addition, $P{2}_{1}$/m-MgZrO_3_ also shows high dielectric constant equals to 313.05, with bandgap of 3.64 eV. Given that DFT calculation systematically underestimates the fundamental bandgap^41^ by 10–40%^15^, it is likely that $P{2}_{1}$/m-MgZrO_3_’s band gap is larger than 4 eV, which can meet the requirement of application of CPU (4 eV) and other CMOS devices.

In the CPU and DRAM industry, dielectrics require $\epsilon$ > 30 for further device scaling^10^. With the additional limitation of *E*_*g*_ > 3 eV for DRAM and *E*_*g*_ > 4 eV for CPU, the qualified materials from our database are listed in Table 5 (materials with calculated dielectric tensor in MP are not included). Most of the materials that meet the requirements are hafnia and zirconium based oxides. To evaluate high-performance dielectric materials for microelectronic device, we applied the fitness model according to Eq. 4. We note that the bandgap can also significantly affect the fitness value, for example, $Pnma$-CaHfO_3_ and $Pmc{2}_{1}$-Sr_3_HfO_5_ have similar dielectric constants (31.05 and 30.05). However, as a result of their bandgap difference (1.12 eV), their fitness values differ dramatically (323.21 for $Pnma$-CaHfO_3_ and 91.06 for $Pmc{2}_{1}$-Sr_3_HfO_5_)

**Table 5:** All materials satisfy $\epsilon  > 30$, *E*_*g*_ > 3 eV for DRAM and *E*_*g*_ > 4 eV for CPU.

Formula	Space group	E above hull	$\epsilon$	*E*_*g*_	fitness	MP-ID
CaHfO_3_	Pnma	0.02	31.05	4.51	323.21	mp-754853
CaHfO_3_	P1	0.08	36.78	4.42	367.67	N/A
CaHfO_3_	Pmc2_1_	0.07	35.36	4.10	304.59	N/A
CaHfO_3_	C2/c	0.04	45.77	4.07	387.86	N/A
SrHfO_3_	Imm2	0.02	93.26	3.72	494.40	N/A
SrHfO_3_	Pmc2_1_	0.02	67.25	3.70	344.07	N/A
Sr_2_HfO_4_	I4/mmm	0.03	81.17	3.69	411.92	N/A
Sr_2_HfO_4_	Pbam	0.06	30.03	3.46	102.87	mp-752537
Sr_3_HfO_5_	Pmm2	0.07	41.37	3.41	129.65	N/A
Sr_3_HfO_5_	Pmc2_1_	0.07	30.05	3.39	91.06	N/A
Ba_3_Hf_2_O_7_	I4/mmm	0.01	32.36	3.58	136.33	mp-754128
BaHfO_3_	Pm $\bar{3}$ m	0.01	39.94	3.55	159.43	mp-998552
Mg_2_ZrO_4_	Pc	0.07	33.18	4.42	331.05	N/A
MgZrO_3_	P2_1_/m	0.06	313.05	3.64	1552.21	N/A
BeZr_6_O_13_	R3	0.02	39.44	4.17	351.72	N/A
BeZr_4_O_9_	C2	0.03	52.98	3.56	216.58	N/A
CaZrO_3_	Pnma	0.00	44.18	4.00	361.19	mp-4571
CaZr_3_O_7_	Pmn2_1_	0.00	57.74	3.51	214.64	N/A
Sr_3_Zr_2_O_7_	I4/mmm	0.00	32.77	3.22	73.02	mp-27690
SrZrO_3_	C2/m	0.00	46.65	3.57	192.30	N/A
SrZrO_3_	Imma	0.00	49.28	3.56	202.18	mp-1080575
SrZrO_3_	Pnma	0.00	40.32	3.73	216.57	N/A
Sr_4_ZrO_6_	C2	0.09	30.71	3.40	94.98	N/A
Sr_3_Zr_5_O_13_	Cm	0.04	35.31	3.37	102.72	N/A
SrZrO_3_	Pmc2_1_	0.00	46.37	3.35	131.94	N/A
SrZrO_3_	Pmc2_1_	0.00	78.20	3.32	209.15	N/A
SrZrO_3_	Cm	0.00	90.88	3.28	225.85	N/A
Sr_2_ZrO_4_	Imm2	0.00	42.03	3.23	94.64	N/A
Sr_3_ZrO_5_	Cm	0.10	63.51	3.20	135.10	N/A
SrZrO_3_	P4mm	0.00	86.31	3.36	248.27	mp-1068742
BaZrO_3_	C2/c	0.00	64.04	3.13	121.14	N/A
Ba_2_ZrO_4_	I4/mmm	0.00	39.86	3.11	71.80	mp-8335
Ga_2_ZrO_5_	C2/m	0.07	31.20	3.01	46.13	N/A

## Technical Validation

**Fig. 5:**
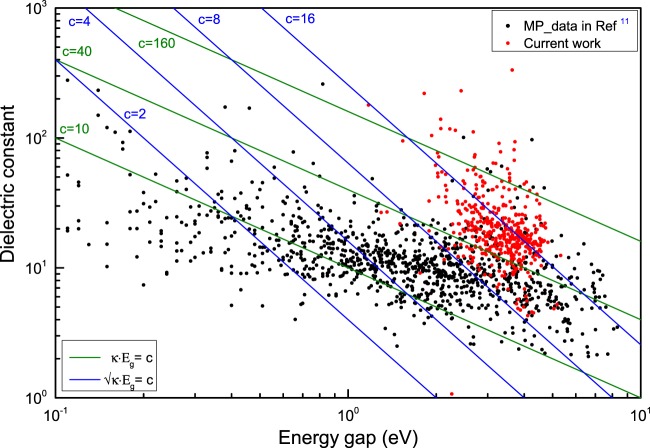
The distribution of high dielectric materials. An updated map for the distribution of band gap and dielectric constants values results based on the results from this work (red) and Petousis *et al*.^11^ (black).

**Fig. 6:**
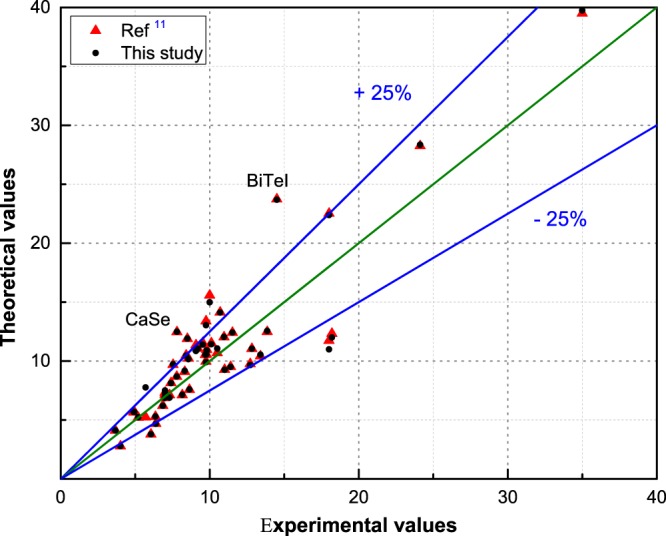
DFPT theoretical versus experimental dielectric constant. A comparison based on the results from this work (black) and Petousis *et al*.^11^ (red). Outliers with a deviation larger than 50% relative to experimental value were marked.

In our calculation, 441 structures of ternary oxides were screened out, as shown in Fig. 5. Comparing with materials data in the Materials Project, our newly generated dataset has both larger bandgap and dielectric constant. Most of the ternary oxides are above $\epsilon \ast {E}_{g}\,=\,40$ and $\surd \epsilon \ast {E}_{g}\,=\,8$. To validate the reliability of our study, we compared the results with Petousis’s work^11^, as shown in Fig. 6, in which they checked the computational error with experiments, and validated the dielectric constants of most materials deviate less than ±25% from experiments at room temperature. Here our study reached the same accuracy. In Fig. 6, most of the dielectric constants in our study (black) and Petousis’ study (red) overlap. We also did a comprehensive comparison by computing the mean absolute error (MAE), root Mean Square Error (RMSE), mean absolute relative deviation (MARD)^12^, Pearson correlation and Spearman rank-order correlation coefficient. The results are: MAE = 2.03, MARD = 19.50%, RMSE = 2.77, Pearson correlation coefficient = 0.90 and Spearman rank-order correlation coefficient is 0.84 with p-value of 2.4 × 10^−14^. Pearson correlation coefficient assesses the linear correlation between two variables and Spearman coefficient is a measure of rank correlation between two sequences of ranking features. Both of these two coefficients are close to 1, indicating that the general consistency between calculation and experiment. We believe our method should be as reliable as the previous work^11^. Given that many factors (e.g., temperature, pressure, impurities, vacancies, interfaces, and surface charges) may cause the theoretical values deviate from experiments^11^. It is reasonable that the theoretical values are not exactly the same with experiment. However, the calculation would still provide an inspiration for further research.

## Usage Notes

In this paper, we employed a first-principles crystal structure prediction method to perform a systematic structural study on a series of ternary oxides systems. For the low energy structures discovered from our prediction, we developed an automated computational screening scheme to evaluate their dielectric and electronic properties. This work generated a library of hypothetical materials which are promising for high dielectric applications. Among them, *P*2_1_/m-MgZrO_3_ achieves the best theoretical performance as it has both large bandgap (3.64 eV) and large dielectric constant (313.05). The rest 32 structures (such as $Pnma$-CaHfO_3_, *Cm*-SrHfO_3_ and $R3$-Zr_6_BeO_13_) with bandgap above 3 eV and dielectric constant above 30, may be useful in CPU or DRAM devices. Their structural, dielectric, and thermodynamic properties are archieved in a supplementary JSON file. In addition, we investigated the factors affecting the dielectric properties, pointing out that the dielectric properties are affected by multi-factors including vibration of atoms, Born effective charge, and atomic mass. Among these newly discovered structures, many of them were predicted from the first principles crystal structure prediction for the first time, suggesting that the crystal structure prediction methods as a complementary approach to the current high throughput screening based on data mining. The computational scheme developed here is entirely general to be used to search for other functional materials as well.

## Data Availability

All reported crystal structure prediction calculations were performed using the USPEX code, which is based on evolutionary algorithms to predict structures with only elemental information^21,42^. Relaxation of structures and DFPT method were carried out by the VASP code^22^.
